# From the Reticuloendothelial to Mononuclear Phagocyte System – The Unaccounted Years

**DOI:** 10.3389/fimmu.2015.00328

**Published:** 2015-07-01

**Authors:** Simon Yona, Siamon Gordon

**Affiliations:** ^1^University College London, London, UK; ^2^Sir William Dunn School of Pathology, The University of Oxford, Oxford, UK

**Keywords:** macrophage, monocyte, Metchnikoff, phagocytosis, dendritic cells, inflammation

## Abstract

It is over 125 years since Ilya Metchnikoff described the significance of phagocytosis. In this review, we examine the early origins and development of macrophage research continuing after his death in 1916, through the period of the reticuloendothelial system. Studies on these cells resulted in a substantial literature spanning immunology, hematology, biochemistry, and pathology. Early histological studies on morphology and *in situ* labeling laid the foundations to appreciate the diversity and functional capacity of these cells in the steady state and during pathology. We complete this phagocyte retrospective with the establishment of the mononuclear phagocyte system nomenclature half a century ago.

## Introduction

The earliest accounts of macrophage research are closely linked with the widespread introduction of the microscope in the mid-nineteenth century, 300 years following the seminal microscopic observations of Antony van Leeuwenhoek (1700) (1). In the histological accounts, von Kölliker (1847) detected cells in the spleen containing particles; later Preyer (1867) observed the internalization of erythrocytes by splenic cells and proposed that this occurred by an active process (2, 3). However, investigators at the time did not associate such observations with a defense mechanism. In fact, Klebs (1872) believed just the opposite, proposing that these cells assist the transport of bacteria to lymphatic tissue (4). Koch (1878) also concluded that these cells provide a suitable microenvironment for bacilli to multiply and disseminate to other tissues, – the so-called Trojan horse theory – after observing numerous bacilli within leukocytes, while studying frogs treated with anthrax (5). Therefore, although cytological observations of the mid-nineteenth century recognized the ability of leukocytes to devour (*fressen*) erythrocytes and microorganisms, opinion at the time did not associate this event with host defense, nor was there a consensus that the process was active or merely the penetration of foreign material into cells to aid infection.

By the late nineteenth century, Metchnikoff (1892), the Russian zoologist and forefather of cellular immunity, established the idea of the phagocyte (6–8). Metchnikoff was the first to fully appreciate the capabilities and purpose of phagocytosis, by performing a series of classical studies spanning from simple unicellular organisms to complex vertebrates. The description of Metchnikoff’s discovery of phagocytosis documented by his wife Olga, now rests in the pantheon of immunology legend.

… One day when the family had gone to see some performing apes at the circus, Metchnikoff with his microscope introduced a rose thorn into the transparent body of a starfish larva, Metchnikoff observed the accumulation of phagocytes surrounding the foreign material and attempting to devour the splinter… (9).

It is important to remember that Metchnikoff started his career as an evolutionary developmental embryologist, influenced by Darwin’s recent publication On the Origin of Species in 1859. As an embryologist, Metchnikoff modeled the early formation of the embryo in primitive organisms, such as sponges, and proposed that an inner “*parenchymella*” contained wandering cells of mesodermal origin capable of taking up particulate matter during embryogenesis. These studies may have been the foundation for Metchnikoff’s phagocytosis theory. Later, Metchnikoff recognized the multiple tasks performed by phagocytosis; as an embryologist, the reabsorption of tissue during embryogenesis, as a zoologist, a common feeding mechanism of unicellular organisms and as a pathologist, its role in host defense. Therefore, when Metchnikoff performed his most notable study, the rose thorn experiment at Messina culminated in the phagocytic process we know today. Metchnikoff was one of, if not the, earliest to demonstrate the evolutionary functional adaptation of a particular biological process, in this case phagocytosis, from a simple feeding mechanism for unicellular organisms, to a developmental requirement during embryogenesis and finally as a necessity for host defense (3, 10).

Metchnikoff’s phagocytes comprised two populations he termed macrophages (large eaters) and microphages (small eaters, later known as polymorphonuclear leukocytes). Contrary to Rudolf Virchow’s impression that inflammation is a continuous life threatening menace, Metchnikoff regarded it as a healing or salutary reaction as postulated 100 years earlier by the Scottish surgeon and collector John Hunter (1794) (11). Therefore, Metchnikoff concluded that the ability of cells to engulf foreign microorganisms acts as an active defense mechanism, giving rise to the concept of cellular innate immunity. At the time, this triggered extensive debate between humoral and cellular schools of thought. Two major events at the turn of the twentieth century helped to reconcile this dispute. First, in 1908, the Nobel Prize in Physiology or Medicine was awarded jointly to Metchnikoff, advocate of the cellularists and to Ehrlich, the champion of humoralist dogmas “*in recognition of their work in immunity*”. Second, in 1903, Wright and Douglas proposed the concept of “opsonization” as a humoral mechanism to increase the susceptibility of bacteria to phagocytosis. These investigators claimed that humoral and cellular functions were not mutually exclusive, rather interdependent (12). This theory was spoofed by George Bernard Shaw, in the introduction to his play “The Doctor’s Dilemma” in 1906.

… Sir Almroth Wright, following up one of Metchnikoff’s most suggestive biological romances, discovered that the white corpuscles or phagocytes, which attack and devour disease germs for us, do their work only when we butter the disease germs appetizingly for them with natural sauce which Sir Almroth Wright named opsonin… (13).

## The Reticuloendothelial System

By the early decades of the twentieth century descriptions of the phagocyte system had become chaotic, not least since the term macrophage had become synonymous with adventitia cell, anode cell, clasmatocyte, dictocyte, erythrophagocyte, histiocyte, polyblast, pyrrhol cell, and rhagiocrine cell; the many terms bestowed on these cells (>30 different names) (14) revealed that the divergence of opinion at the time as to the relationship of these cells to one another and from tissue-to-tissue. Not only were tissue phagocytes given a variety of bewildering names but also their origin remained unknown. From time-to-time, historic discoveries are lost in the ether of *a priori* thought; this is certainly true for histological techniques that assisted in the classification of blood cytology. Until Ehrlich’s early effort to develop leukocyte cytological staining, scholars of blood operated solely on fresh samples. Ehrlich’s aim was to take advantage of the known chemical structures of dyes and their interaction with cellular bodies to map and characterize the anatomy of blood cells. By using aniline dyes in combination with neutral dyes and the morphology of the nucleus, he was able to divide cells of the blood into mononucleated lymphocytes, some of which were large, large mononuclears with indented nuclei (now known as monocytes) and polymorphonuclear cells that were neutrophilic (neutrophils), acidophilic (eosinophils), or basophilic granules (basophils/mast cells) (15). By the early twentieth century, Ribbert (1904) had performed studies with lithium carmine solution injected into the peripheral circulation and observed the specific uptake and storage by a group of cells, which became *vitally stained* (16). These were subsequently demonstrated to be mononuclear cells phagocytosing particulate matter. Clark and Clark (17) described these large mononuclear cells in tissues to be the same as “clasmatocytes,” described by Louis-Antoine Ranvier, the “Polyblasts” of Alexander Maximow and the “Histiocytes” defined by Kenji Kiyono. Following these early observations, it became apparent that a large number of histological dyes including trypan blue, neutral red, isamine blue, and other colloids discriminated phagocytes from fibroblasts. The systematic analysis of tissues and dyes led Karl Albert Ludwig Aschoff (1924) to coin the term “reticuloendothelial system” (RES) to describe this group of cells, with their ability to incorporate vital dyes from the circulation (18). Reticulo refers to the propensity of these large phagocytic cells in various organs to form a network or a *reticulum* by cytoplasmic extensions; *endothelial* refers to their proximity to the vascular endothelium (19), from which they were sometimes believed to arise, these cells formed Aschoff’s unified system throughout the organism. The capture and clearance of unwanted particulate material from blood and lymph were considered to be the major function of the RES. Although opinions about the origin of cells of the RES will be discussed later in this series; at the time, Aschoff considered that the cells of the RES were derived locally and that both histiocytes and reticulum cells shared a common origin.

## Cells of the Reticuloendothelial System

Metchnikoff had previously described the dissemination of macrophages throughout the organism and Aschoff’s system implied a common function of the cells of the RES even in the absence of inflammation. In the next section, we highlight some of the tissue locations and possible functions assigned at the time.

### Kupffer cells

The macrophages of the liver are located within the sinusoids, which is composed of four cell types, each with its own morphology and function. Karl Wilhelm von Kupffer (1876) observed “*Sternzellen*” (star cells) in the liver and believed them to be an integral part of the hepatic endothelium. Later, Tadeusz Browicz (1899) identified Kupffer’s cells as the phagocytes of the liver (20) (sometimes known as Browicz–Kupffer cells) and observed that they could take-up a large percentage of vital stain. In the early 1930s, Peyton Rous developed an ingenious method to isolate Kupffer cells of the liver. Rous and Beard injected a suspension of gamma ferrous oxide i.v., light in weight but highly magnetic particles, Kupffer cells efficiently phagocytosed these particles. They then perfused and processed the liver and the phagocytes were then selected by magnetic force, to the best of our knowledge the first description of magnetic cell sorting (21, 22), enabling the extraction of macrophages from a solid tissue for examination *in vitro*. The origin of Kupffer cells like all cells of the RES at the time remained a source of continued confusion and debate. At the American Association of Anatomists conference in 1925, M. R. Lewis presented a paper comparing Kupffer cells isolated from frogs, thought to be derived from endothelial cells, side-by-side with an examination of clasmatocytes and concluded these cells were identical in morphology and function (23).

In 1950s, Baruch Benacerraf, Nobel laureate in 1980 for his work on MHC with George Snell and Jean Dausset (24), teamed up with Guido Biozzi, a young Italian in the Halpern laboratory in Paris in a productive collaboration. They developed techniques to study clearance of particulates from blood and formulated equations that govern this in mammals. In subsequent work, Biozzi bred strains of mice differing in the quantitative antibody response to various antigens. Biozzi mice are still in use to study autoimmune inflammatory neurological disease (25). These studies in mice and guinea pigs helped to introduce genetic approaches to the role of macrophages in innate and adaptive immunity.

## Microglia and the Origin of the RES

Virchow (1858) acknowledged that the central nervous system (CNS) was composed of both neurones and interstitial cells, which he termed neuroglia (26, 27). By the end of the nineteenth century, the Scottish pathologist William Ford Robertson confirmed that neuroglia were indeed composed of multiple cell types (28). Robertson continued to investigate this heterogeneous population of cells; with the aid of platinum staining techniques he was able to distinguish a novel cell type in the brain he termed mesoglia (as he believed that they were mesodermal in origin). Finally, Robertson deduced that mesoglia possessed phagocytic properties (29). In fact, Robertson had identified oligodendroglia and mesodermal derived cells, under the term mesoglia. In 1913, Santiago Ramon y Cajal described a group of cells derived from the mesoderm as the “*third element*” of the CNS, the first element being neurones and the second element the astrocytes, derived from ectoderm. It was the Spanish pathologist Pio Del Rio-Hortega who revolutionized our understanding of neuroglia from a series of detailed studies using silver carbonate impregnation staining. He uncovered a homogeneous group of cells within the CNS with tree like projections and predicted that they possess phagocytic functions within the CNS; he termed these cells as microglia (26, 27). Hortega laid the groundwork for microglia research in a lecture given at University of Oxford, microglia enter the CNS during development from mesodermal origin where they disseminate throughout the CNS and take-up a branched ramified cytological appearance. He went on to explain that they remain evenly spaced in the steady state, while pathological insults cause microglia to take on an amoeboid morphology, express the ability to phagocytose and to migrate (30). These studies confirmed that the microglia of the CNS belonged to the RES. The account he gave in Oxford remains accurate to this day. Interestingly, although microglia were unable to take-up vital dyes because of the blood brain barrier, they were known to be highly phagocytic, reticuloendothelial cells readily stained by silver carbonate. Although Hortega described microglia to be derived from cells of the mesoderm during embryogenesis, this still remained a matter of great debate, until recently. Early observations in the late nineteenth and several studies in the early twentieth described microglia during neurodegenerative diseases without a clear understanding of their origin.

### Osteoclasts

The histological identification of a cell that resorbs bone can be traced back to the early 1850s. Tomes and De Morgan (31) described within a section of diseased femur, cavities that were associated with an increase in nucleated cells (31). By 1873, Kölliker described multinucleated giant cells involved in bone absorption that he termed *Ostoklast* and anticipated that these cells are involved in homeostatic and pathological bone degradation (32, 33). The notion of a bone-resorbing cell became widely accepted (34). Over the next 50 years, the morphology of the osteoclast was refined and interestingly these large multinucleated cells showed variation in size and nuclear content; in pigs, they contained as many as 100 nuclei (35) while human osteoclasts could contain up to 50 nuclei (32). John Loutit an Australian born pioneer in radiation biology from the late 1940s studied not only osteoclast origin from blood precursors but also the biology of bone marrow transplantation after irradiation (36) in a long and productive career at Harwell MRC laboratories.

### Alveolar macrophages and phagocytosis

The lung also contains many mononuclear phagocytes, which are associated with the alveoli and the alveolar space (37). The macrophages within the alveolar space were initially known as “dust cells” because of their content of intracellular carbon particles. There is a constant requirement to keep the alveolar space free of particles and pathogens allowing for optimal oxygen transfer, the major role of these cells. As the lung occupies a unique accessible position among internal organs, it is constantly in contact with the external world. In the late 1950s, Karrer observed the efficient phagocytosis of India ink exclusively by free alveolar macrophages, similar to the previous observations of increased carbon particles in these cells of city dwellers (38, 39). The question of the origin of the macrophage troubled cytologists and immunologists for most of the twentieth century; this was no different in the lung.

One of the best-studied pathologies in the first half of the twentieth century in relation to macrophages was pulmonary tuberculosis. The initial stage of tuberculosis displays the transient influx of neutrophils described by Maximow in 1925 (40). However, these cells are unable to destroy the bacilli and monocyte/macrophages remain the most prominent infected host cells. From 1920s, Sabin, the first full female Member of the Rockefeller Institute and first female elected to the National Academy of Sciences, considered the monocyte response to tuberculosis the most significant process, “*cellular and immunological reactions in tuberculosis center around the monocyte*,” when she first proposed this she was mocked by her peers. Sabin observed monocytes to become epithelioid cells that develop into macrophage giant cells (41), previously described by the German pathologist Theodor Langhans as a hallmark of tuberculous granulomata already in the nineteenth century. In 1930s, Max Lurie, an advocate of the monocyte theory, used inbred rabbits to study their susceptibility to bovine tuberculosis. Lurie observed resistant inbred rabbits went on to develop cavitary tuberculosis while susceptible families went on to develop disseminated tuberculosis (42). The Australian immunologist, George Mackaness studied the role of anti-TB drugs on infected macrophages when a student at the Sir William Dunn School of Pathology, University of Oxford with Howard Florey in the early 1950s. His subsequent studies in the sixties at the Trudeau Institute in Saranac Lake defined T lymphocyte-dependent activation of macrophages by BCG and *Listeria* infection (43, 44), Mackaness coined the term macrophage activation, the so-called “angry” macrophages (45). Dannenberg has been another pioneer of macrophage research in experimental and clinical tuberculosis, especially in the characterization of the granuloma (46).

Other resident phagocytic populations were described in many tissues during this period, for example, in the skin (Langerhans cells), gut, lympho-hemopoietic tissues, reproductive and endocrine organs, and placenta (Hoffbauer cells). We draw attention to specialized macrophages in bone marrow stroma, where they appear at the center of hematopoietic islands, first described in detail by Marcel Bessis and his collaborators (47). John Humphrey drew attention to the marginal metallophilic macrophages located in a zone between the red and white pulp of spleen, especially in rodents (48, 49). They line a sinusoidal space where they sample circulating blood for viruses, for example, and play an important role in clearance of T cell-independent immunogenic polysaccharides. Tingible body macrophages (TBM) were identified by Walther Flemming in 1885; located in germinal centers. TBM contain phagocytosed apoptotic cell debris (tingible bodies) and are involved in the clearance of apoptotic lymphocytes (50), these observations were confirmed by electron microscopy in the early 1960s (51). Finally, the peritoneal macrophages of the mouse, responsible for much of our knowledge of macrophage immunobiology, were first described as a tractable cell population by Cohn only in 1962 (52).

## The Origin of Macrophages

As Aschoff was formulating the requirements of the RES, a number of research groups were searching for the origin of macrophages. In 1914, Awrorow and Timofejewskij concluded from the outgrowth of leukocytes from leukemic blood that the lymphocyte is the progenitor from which macrophages arise (53). A few years later, several *in vitro* studies described the differentiation of blood monocytes into macrophages (53–55); Carrel and Ebeling (55) and Lewis and Lewis (23) observed that blood cultures over time developed into macrophages that had phagocytosed the debris of other blood cells, concluding that these monocyte-derived cells became actively phagocytic and were indistinguishable from macrophages in staining with neutral red (54, 55). At the same time, in 1925, Sabin took a cytological approach using neutral red staining to examine resident macrophage populations in connective tissue (clasmatocytes), concluding that a proportion of macrophages were derived from bone marrow-derived monocytes (56). However, the first *in vivo* study to examine how mammalian blood monocytes behave during an acute pathological insult was performed by Ebert and Florey (57) at the University of Oxford, using the rabbit ear chamber, observing diapedesis of blood monocytes toward the site of tissue injury. These monocytes transformed into macrophages during the inflammatory process; they concluded “*The cells originating from monocytes eventually became cells which we are calling histiocytes, which are indistinguishable from the so-called resting wandering tissue-cell of Maximow*” (57). Twenty-five years later, Volkman and Gowans (58) confirmed these findings using thymidine autoradiography and parabiosis inferring that macrophage precursors are rapidly dividing cells derived from a remote site during inflammation (58). Takahashi mentions in his comprehensive review on macrophage ontology how the Japanese pathologist Amano with the aid of supravital staining at the inflammatory foci observed blood monocytes to be precursors of the macrophage (59). Finally, Marchesi and Florey employing electron microscopy were able to distinguish the earliest phase of monocyte migration during mild inflammation, which occurred during the maximal efflux of neutrophils (14, 60). The conclusion from these studies suggests that macrophages derived from circulating monocytes were based on inflammatory models. Therefore, a more accurate conclusion would be that during inflammation monocytes become effector cells by concentrating at the site of injury with the ability to produce large quantities of inflammatory mediators (61).

Another important and well-studied population of recruited monocyte-derived cells are the foam cells, a hallmark of atheromatous pathology. A pupil of Maximow in St. Petersburg and later the student of Aschoff in Freiburg (62), Anitschkow (63, 64) showed that simply by feeding rabbits purified cholesterol caused vascular changes leading to the formation of lesions similar to those seen during atherosclerosis in humans (63, 64). Anitschkow decided that these cholesterol-laden cells were of leukocyte origin (65). Anitschkow’s work on lipid storage was compared to the work by Robert Koch on the tubercle bacillus (66). It was mainly as a result of the work by Russell Ross in 1970s that these monocyte-derived cells have been categorized as part of a fat modified inflammatory process (67). Recruited monocytes can also give rise to multinucleated giant cells, not only a feature of tuberculosis. They are found in many granulomatous inflammatory diseases, including viral and parasitic infections, and in responses to foreign bodies and fat necrosis (Touton cells), named after the German dermatologist Karl Touton (1885) (68).

The accumulation of data and the introduction of new techniques highlighted that the cells of the RES differ in morphology, function, and origin (14). In addition, the underlying processes involved in these functional and morphologic alterations remained unknown. Is there a proliferating mononuclear phagocyte population within the RES, constantly differentiating in the steady state? These questions continued to puzzle scientists throughout the twentieth century.

## The Mononuclear Phagocyte System

… The most immature cell of the mononuclear phagocyte system … is the promonocyte … that by dividing gives rise to monocytes … Monocytes in the circulation constitute a mobile pool of relatively immature cells on their way from the place of origin to the tissues. At sites where conditions are favourable for phagocytosis, these cells become macrophages… (69).

**Figure 1:**
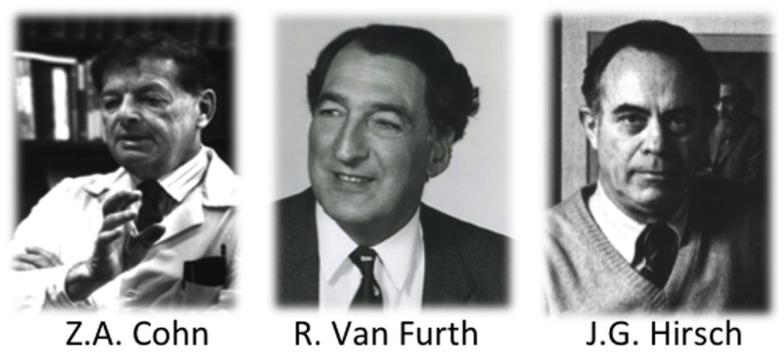
**Selected experimental pathologists and immunologists who coined the term mononuclear phagocyte system**.

As knowledge accumulated, the term RES was regarded as insufficient to describe resident phagocytes and their antecedents. At a scientific meeting in Leiden in 1969, a group of prominent pathologists/immunologists proposed the term “mononuclear phagocyte system” (MPS) as a more accurate term (Figure 1) (69). The MPS at the time comprised monocytes and macrophages derived from the bone marrow derived monocytes. Nevertheless, little evidence existed to suggest that monocytes differentiate into resident macrophages under steady state conditions. Interestingly, Maximow proposed on the basis of embryonic studies on amphibians and mammals that macrophages and leukocytes may arise from distinct lineages (70).

While the MPS was being formulated in 1960s, immunologists were in pursuit of the “*third cell*” (71) a requirement for adaptive-immune responses. Steinman and Cohn in their landmark study (1973) identified and characterized the dendritic cell (DC) as distinct from macrophages (72, 73); over time, the DC became accepted as the third arm in the trinity we know today as the MPS (74). Monocytes, macrophages, and DCs are distinguished on the basis of morphology, function, and origin, yet collectively constitute the MPS.

As more data accumulated in the early decades of the twenty-first century, it emerged that most tissue macrophage populations in adults in the steady state are maintained independent of the bone marrow and rely almost exclusively on self-renewal (75, 59, 76–86). These data facilitated the reexamination of the concept of the MPS (87).

## Conclusion

We have highlighted only a few of the many milestones of macrophage biology from its early origins to the establishment of the MPS nomenclature in 1968. Studies during this period resulted in a substantial literature spanning immunology, hematology, and pathology. A number of important issues emerge from a retrospective analysis of the literature. First, we learn that rarely in science do revolutions occur from a single Eureka moment rather years of observation culminate in new findings. While Metchnikoff’s phagocytosis theory seems to have emerged from his experiments on starfish larvae, he had previously observed cells capable of taking up particulate matter during embryogenesis (10). Second, the first half of the twentieth century, blighted by two World Wars, had profound impacts on science, resulting in a geographical and common language shift of scientific research. Third, the techniques used routinely in the laboratory shifted from the pathologists’ tool box of the microscope and microbiology to the immunologists’ introduction of cell transfer, thymidine autoradiography, immunohistochemistry, parabiosis, electron microscopy, later flow cytometry, cell, and molecular biology. However, if one was able to transport Metchnikoff, Aschoff, or Cohn to a conference in 2015 on mononuclear phagocytes they would perhaps not appreciate the cytokines, chemokines, blots, and plots; however, the fundamental questions and discussions remain familiar; what is the origin of these cells? How do they phagocytose? Do macrophages proliferate *in situ*? How is particulate material recognized and cleared? This is why it is important to examine the history of our field since our research questions today are more closely linked to the past than we may appreciate.

The macrophage story is not over. In recent months, further strides have been made in examining the molecular signatures, characterizing the MPS in the steady state and upon enhanced recruitment of monocytes during inflammation (88–94). These studies highlight collective attributes of the macrophages; however, they also show significant local adaptations associated with particular functions within a specific organ. The next stage on this journey will include recreating *in vitro* the phenotypes of these specific populations using induced pluripotent stem cells, and gaining a greater insight into how these cells behave under steady state conditions *in situ*, as well as during and after the inflammatory response. Finally, the role of the circulating monocyte is also undergoing a re-evaluation; previously, monocytes were viewed as the bridge from bone marrow progenitors to fully differentiated tissue macrophages not only after inflammation, injury, and infection but also for resident populations in the absence of inflammation, as stated in Van Furth’s description of the MPS “*Monocytes in the circulation constitute a mobile pool of relatively immature cells on their way from the place of origin to the tissues*” (69). Moreover, monocytes should now be further investigated as distinct precursors of only newly recruited monocyte-derived cells and as effector cells in their own right.

## Conflict of Interest Statement

The authors declare that the research was conducted in the absence of any commercial or financial relationships that could be construed as a potential conflict of interest.
